# Monitoring of serum lactate level during cardiopulmonary resuscitation in adult in-hospital cardiac arrest

**DOI:** 10.1186/s13054-015-1058-7

**Published:** 2015-09-21

**Authors:** Chih-Hung Wang, Chien-Hua Huang, Wei-Tien Chang, Min-Shan Tsai, Ping-Hsun Yu, Yen-Wen Wu, Kuan-Yu Hung, Wen-Jone Chen

**Affiliations:** Department of Emergency Medicine, National Taiwan University Hospital Yunlin Branch, Yunlin, Taiwan; Graduate Institute of Clinical Medicine, College of Medicine, National Taiwan University, Taipei, Taiwan; Department of Emergency Medicine, National Taiwan University Hospital and National Taiwan University College of Medicine, No.7, Zhongshan S. Rd., Zhongzheng Dist., Taipei City, 100 Taiwan R.O.C.; Department of Emergency Medicine, Taipei Hospital, Ministry of Health and Welfare, New Taipei City, Taiwan; Departments of Internal Medicine and Nuclear Medicine, National Taiwan University Hospital and National Taiwan University College of Medicine, Taipei, Taiwan; Department of Nuclear Medicine and Cardiology Division of Cardiovascular Medical Center, Far Eastern Memorial Hospital, New Taipei City, Taiwan; National Yang-Ming University School of Medicine, Taipei, Taiwan; Division of Nephrology, Department of Internal Medicine, National Taiwan University Hospital, Taipei, Taiwan; Department of Emergency Medicine, Lotung Poh-Ai Hospital, Yilan, Taiwan

## Abstract

**Introduction:**

Serum lactate level may correlate with no-flow and low-flow status during cardiac arrest. Current guidelines have no recommended durations for cardiopulmonary resuscitation (CPR) before transition to the next strategy. We hypothesized that the lactate level measured during CPR could be associated with the survival probability and accordingly be useful in estimating the optimal duration for CPR.

**Methods:**

We conducted a retrospective observational study in a single medical centre and included adult patients who had suffered an in-hospital cardiac arrest between 2006 and 2012. We used multivariable logistic regression analysis to study the association of lactate level measured during CPR and outcomes. We used generalized additive models to examine the nonlinear effects of continuous variables and conditional effect plots to visualize the estimated survival probability against CPR duration.

**Results:**

Of the 340 patients included in our analysis, 50 patients (14.7 %) survived to hospital discharge. The mean lactate level was 9.6 mmol/L and mean CPR duration was 28.8 min. There was an inverse near-linear relationship between lactate level and probability of survival to hospital discharge. A serum lactate level <9 mmol/L was positively associated with patient survival to hospital discharge (odds ratio 2.00, 95 % confidence interval 1.01-4.06). The optimal CPR duration may not be a fixed value but depend on other conditions.

**Conclusions:**

Serum lactate level measured during CPR could correlate with survival outcomes. A lactate level threshold of 9 mmol/L may be used as a reference value to identify patients with different survival probabilities and determine the optimal CPR durations.

**Electronic supplementary material:**

The online version of this article (doi:10.1186/s13054-015-1058-7) contains supplementary material, which is available to authorized users.

## Introduction

More than 200,000 hospitalized adult patients experience in-hospital cardiac arrest (IHCA) annually in the USA with an estimated incidence of 1.6 per 1,000 hospital admissions [1]. The survival rate from IHCA has increased over the past decade [2], probably due to earlier recognition of cardiac arrest, higher quality of cardiopulmonary resuscitation (CPR), and improved post-resuscitation care [3, 4]. Despite this progress, mortality following IHCA remains high, with only about 18 % of patients surviving to hospital discharge [1].

The causes of high mortality could be due to the inability to establish rapid return of spontaneous circulation (ROSC) leading to subsequent multi-organ failure. Extracorporeal CPR (ECPR) has been advocated as a novel alternative for cardiac arrests that are considered refractory to initial conventional CPR [5, 6], especially for IHCA [7]. Nevertheless, the term, “refractory cardiac arrest,” is ill-defined. Most studies on ECPR have used CPR duration as an indicator of futile resuscitation, which varied from 10 to 15 minutes [7–9] and led to initiation of ECPR within 30 to 60 minutes of CPR.

However, using CPR duration alone may not be an accurate indicator for estimating survival probability and determination of futile CPR. Matos et al. [10] reported that even in the case of equivalent CPR durations, the survival probability still differed greatly between different patient groups. Although studies have suggested that the greater the time prior to initiation of ECPR, the poorer the outcome [7], few studies have been carried out on the optimal transition time to consider alternative resuscitation methods in patients with potentially prolonged CPR. Therefore, finding additional parameters besides CPR duration to assist clinicians in more accurately estimating the survival probability during CPR could help strike a balance between optimizing patient outcomes and avoiding the misuse of scarce critical care medical resources, such as ECPR.

Weil et al. [11] found that serum lactate level measured within 10 minutes of CPR could serve as a sensitive indicator of prognosis. A higher post-ROSC serum lactate level [12–16] or a slower clearance rate of serum lactate after ROSC [12, 17, 18] has also been shown to correlate with poor outcomes after cardiac arrest. We therefore hypothesized that the serum lactate level measured during CPR may be associated with the survival probability and accordingly be useful in estimating the optimal CPR duration before transition to the next strategy for IHCA.

## Methods

### Setting

We carried out a retrospective cohort study in a tertiary medical centre, National Taiwan University Hospital (NTUH). Before data collection, the Research Ethics Committee of the National Taiwan University Hospital approved this study (reference number: 201505161RIN) and waived the requirement for written informed consent. The NTUH has 2,600 beds, including 220 beds in intensive care units (ICUs). According to hospital policy, a code team is activated when cardiac arrest events occur in the general wards. Each code team member has been certified to provide advanced cardiac life support. For cardiac arrest events in the ICUs, resuscitation is performed by the staff of the ICU where the cardiac arrest events have occurred and by staff from neighbouring ICUs.

### Participants

We included patients who had experienced an IHCA at the NTUH between 2006 and 2012. We used the following inclusion criteria: (1) age ≥18 years; (2) documented absence of pulse with performance of chest compression for ≥2 minutes; (3) without do-not-resuscitate order; and (4) serum lactate level measured during the initial 10 minutes of CPR. If multiple cardiac arrest events occurred in a single patient, we only recorded the first event of the same hospitalization. We excluded patients who had suffered a cardiac arrest related to major trauma.

### Data collection and outcome measures

We recorded the following information for each patient: demographics, comorbidities, variables derived from the Utstein template [19], the first serum lactate level measured during CPR, and any critical intervention that was implemented. CPR duration was defined as the time from the first chest compression provided by the code team or ICU members to the termination of resuscitation efforts, either due to ROSC or declaration of death. The definitions of comorbidities are appended in Additional file 1: Table S1.

The primary outcome was survival to hospital discharge. Secondary outcomes included 10-minute ROSC, defined as ROSC within 10 minutes of CPR, and favourable neurological status at hospital discharge, defined as a score of 1 or 2 on the cerebral performance category (CPC) scale [20]. The CPC score was retrospectively determined by reviewing medical records for each patient.

### Statistical analysis

We used R 2.15.3 software (R Foundation for Statistical Computing, Vienna, Austria) for data analysis. Categorical data were expressed as counts and proportions; continuous data were expressed as means and standard deviations. Categorical variables were compared by the Fisher’s exact test, and continuous variables were examined by the Wilcoxon rank-sum test. A two-tailed *p* value of ≤0.05 was considered statistically significant.

We selected the odds ratio as the outcome measure. We conducted a multivariable logistic regression analysis to examine the association between independent variables and outcomes. All available variables were considered in the regression model, regardless of whether they were significant by univariate analysis. The stepwise variable selection procedure (with iterations between the forward and backward steps) was applied to obtain the final regression model. Significance levels for entry and for stay were set at 0.15 to avoid exclusion of potential candidate variables. The final regression model was identified by excluding individual variables with a *p* value >0.05, until all regression coefficients were statistically significant. If the lactate level was excluded during the variable selection procedure, we would re-enter it into the final model to obtain the effect estimate.

We used generalized additive models (GAMs) [21] to examine the nonlinear effects of continuous variables and, if necessary, to identify the appropriate cutoff point(s) for dichotomizing a continuous variable during the variable selection procedure. We used conditional effect plots to visualize the predicted probability of survival to hospital discharge against CPR duration, while maintaining the other independent variables in the final model constant. Based on the conditional effect plot, we used CPR duration of 30 minutes and probability of survival of 18 % [1] as reference to calculate the corresponding probabilities of survival and CPR duration, respectively, for each group of patients.

We assessed the goodness-of-fit of the fitted regression model using *c* statistics (the estimated area under the receiver operating characteristic curve), adjusted generalized *R*^2^, and the Hosmer-Lemeshow goodness-of-fit test.

## Results

A total of 1,123 adult patients received chest compression ≥2 minutes in the period between 2006 and 2012. Of these, only 349 patients had concomitant measurements of serum lactate level during the initial 10 minutes of CPR. Nine patients were excluded due to trauma-related cardiac arrest. The remaining 340 patients were included for further analysis.

The characteristics of the patients included in this study, stratified by primary outcome, are outlined in Tables 1 and 2. The mean age of the patients was 65.9 years. A total of 151 cardiac arrest events (44 %) occurred in the ICUs and 171 cardiac arrest events (50 %) occurred in the general wards. Shockable rhythms represented 14 % of initial arrest rhythms. The mean lactate level was 9.6 mmol/L and the mean CPR duration was 28.8 minutes. Both lactate levels and CPR durations were significantly different between survivors and non-survivors. There were 242 patients (71 %) who achieved ROSC and 76 of these patients (22 %) achieved a 10-minute ROSC. Only 50 patients (15 %) survived to hospital discharge and 21 of these patients (6 %) displayed a favourable neurological status.

**Table 1:** Baseline characteristics of study patients stratified by survival outcome

Variables	All patients	Patients surviving to hospital discharge	Death at hospital discharge	*P* value
	(n = 340)	(n = 50)	(n = 290)	
Age, y, mean (SD)	65.9 (16.2)	65.2 (16.2)	66.0 (16.2)	0.75
Male, n (%)	214 (63)	37 (74)	177 (61)	0.08
Comorbidities, n (%)				
Heart failure	93 (27)	17 (34)	76 (26)	0.30
Myocardial infarction	45 (13)	9 (18)	36 (12)	0.27
Arrhythmia	55 (16)	11 (22)	44 (15)	0.22
Hypotension	68 (20)	8 (16)	60 (21)	0.57
Respiratory insufficiency	236 (69)	34 (68)	202 (70)	0.87
Renal insufficiency	138 (41)	22 (44)	116 (40)	0.64
Hepatic insufficiency	64 (19)	4 (8)	60 (21)	0.03
Metabolic or electrolyte abnormality	66 (19)	9 (18)	57 (20)	1
Diabetes mellitus	112 (33)	23 (46)	89 (31)	0.05
Baseline evidence of motor, cognitive, or functional deficits	63 (19)	12 (24)	51 (18)	0.32
Acute stroke	16 (5)	3 (6)	13 (5)	0.71
Favorable neurological status 24 hours before cardiac arrest	143 (42)	25 (50)	118 (41)	0.28
Pneumonia	111 (33)	19 (38)	92 (32)	0.42
Metastatic cancer or any blood-borne malignancy	68 (20)	6 (12)	62 (21)	0.18

*SD* standard deviation

**Table 2:** Features of cardiac arrest events stratified by survival outcome

Variables	All patients	Patients surviving to hospital discharge	Death at hospital discharge	*P* value
	(n = 340)	(n = 50)	(n = 290)	
Arrest at night, n (%)	218 (64)	29 (58)	189 (65)	0.34
Arrest at the weekend, n (%)	105 (31)	17 (34)	88 (30)	0.62
Arrest location, n (%)				0.69
Intensive care unit	151 (44)	23 (46)	128 (44)	
General ward	171 (50)	26 (52)	145 (50)	
Others	18 (5)	1 (2)	17 (6)	
Witnessed arrest, n (%)	241 (71)	35 (70)	206 (71)	0.87
Monitored status, n (%)	199 (59)	28 (56)	171 (59)	0.76
Shockable rhythm, n (%)	49 (14)	14 (28)	35 (12)	0.007
Critical care interventions in place at time of arrest, n (%)				
Mechanical ventilation	64 (19)	7 (14)	57 (20)	0.44
Anti-arrhythmics	30 (9)	5 (10)	25 (9)	0.79
Vasopressors	154 (45)	16 (32)	138 (48)	0.05
Dialysis	23 (7)	2 (4)	21 (7)	0.55
Pulmonary artery catheter	2 (0.6)	2 (4)	0 (0)	0.02
Intra-aortic balloon pumping	1 (0.3)	0 (0)	1 (0.3)	1
Lactate, mmol/L, mean (SD)	9.6 (4.6)	7.2 (4.1)	10.0 (4.5)	<0.001
CPR duration, minutes, mean (SD)	28.8 (26.9)	12.4 (12.8)	31.6 (27.7)	<0.001
Post-ROSC interventions, n (%)				
Extracorporeal membrane oxygenation	20 (6)	3 (6)	17 (6)	1
Therapeutic hypothermia	1 (0.3)	0 (0)	1 (0.3)	1
Percutaneous coronary intervention	8 (2)	3 (6)	5 (2)	0.10

*SD* standard deviation, *CPR* cardiopulmonary resuscitation, *ROSC* return of spontaneous circulation

All variables listed in Tables 1 and 2 were included in the variable selection procedure for the primary outcome. The GAM plot revealed a near-linear association between logit (*p*), where *p* represented the probability for survival to hospital discharge, and lactate level (Additional file 2: Figure S1). If logit (*p*) was ≥0, the odds for survival would be ≥1. Therefore, 9 mmol/L of lactate was selected as the cutoff point to transform the lactate level into a binary variable. The characteristics and outcomes of patients in this study, stratified by lactate level, are reported in Additional file 3: Table S2, Additional file 4: Table S3, and in Table 3.

**Table 3:** Outcomes after cardiac arrest stratified by lactate levels

Variables	All patients, (n = 340) Number (%)	Lactate level <9 mmol/L, (n = 147) Number (%)	Lactate level ≧9 mmol/L, (n = 193) Number (%)	*P* value
ROSC	242 (71)	109 (74)	133 (69)	0.33
ROSC within 10 minutes of CPR	76 (22)	48 (33)	28 (15)	<0.001
ROSC >20 minutes	201 (59)	95 (65)	106 (55)	0.08
Survival >24 hours	129 (38)	76 (52)	53 (28)	<0.001
Survival to hospital discharge	50 (15)	34 (23)	16 (8)	<0.001
Favourable neurological outcome at hospital discharge	21 (6)	16 (11)	5 (3)	0.002

*ROSC* return of spontaneous circulation, *CPR* cardiopulmonary resuscitation

**Fig. 1:**
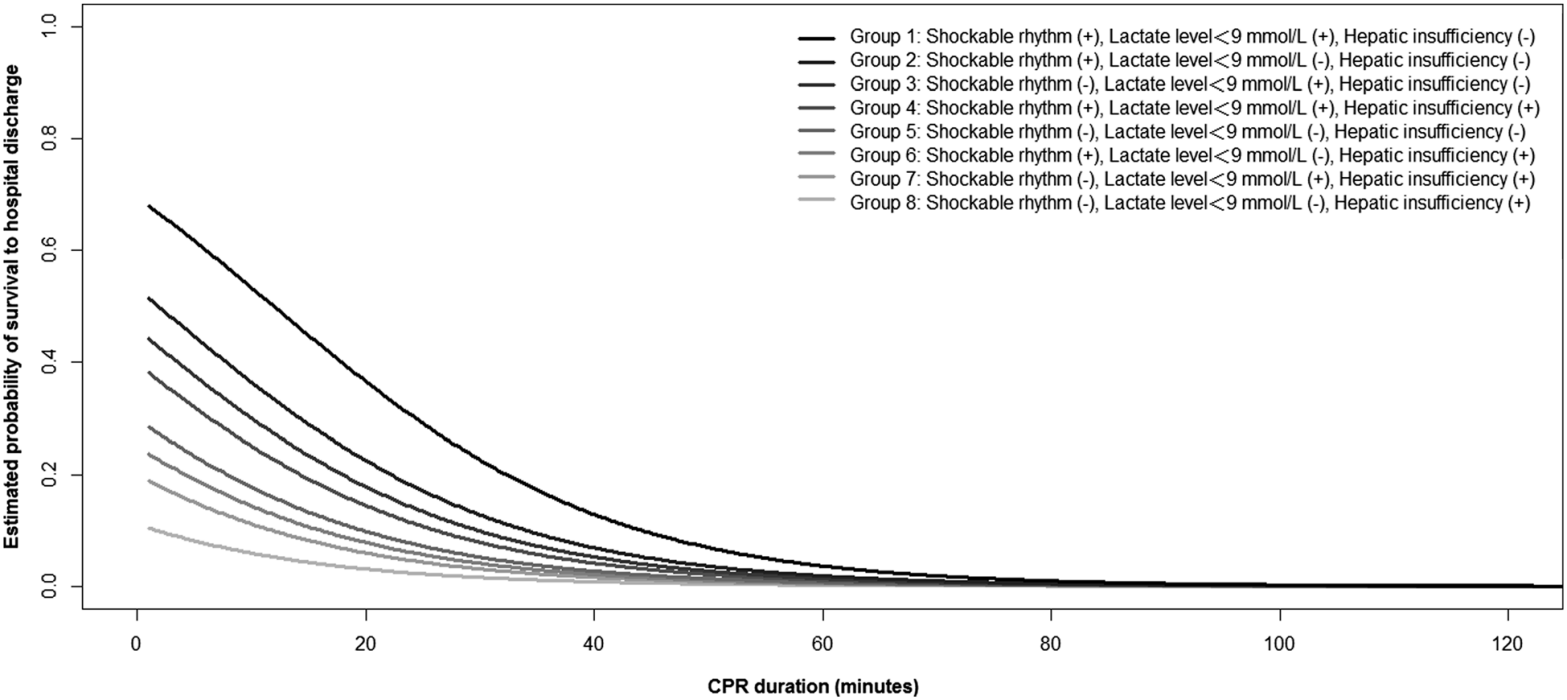
Conditional effect plot of duration of cardiopulmonary resuscitation (*CPR*) on the estimated probability of survival to hospital discharge. The plot was based on the multiple logistic regression model for survival to hospital discharge

The final regression model is shown in Table 4. Shockable rhythm/lactate level <9 mmol/L was positively associated with survival to hospital discharge, and hepatic insufficiency/CPR duration was inversely associated with survival to hospital discharge. The association between CPR duration and estimated survival probability for different patient groups is illustrated in Fig. 1. With reference to Fig. 1, we calculated the survival probability when CPR was performed for 30 minutes for each patient group. We also calculated the CPR duration when survival probability dropped to 18 % or when survival probability was half the initial probability (Table 5).

**Table 4:** Multiple logistic regression model with survival to hospital discharge as the dependent variable

Independent variable	Odds ratio	95 % Confidence interval	*P* value*
CPR duration	0.93	0.90, 0.96	<0.001
Shockable rhythm	2.67	1.18, 5.94	0.02
Hepatic insufficiency	0.29	0.08, 0.82	0.03
Lactate level < 9 mmol/L	2.00	1.01, 4.06	0.05

*CPR* cardiopulmonary resuscitation

*The display of independent variables is arranged in order of *P* value.

**Goodness-of-fit assessment: n = 340, adjusted generalized *R* ^*2*^ = 0.276, the estimated area under the receiver operating characteristic curve = 0.817, and the Hosmer and Lemeshow goodness-of-fit chi-squared test *p* = 0.19

**Table 5:** Probability of survival to hospital discharge and duration of cardiopulmonary resuscitation for different patient groups according to the regression model

Patient groups divided by variables identified in the regression model	Probability of survival to hospital discharge when CPR was performed for 30 minutes	Duration of CPR when probability of survival to hospital discharge decreased to 18 % (minutes)	Duration of CPR when probability of survival to hospital discharge was half the initial probability (minutes)
Group 1: shockable rhythm (+), lactate level <9 mmol/L (+), hepatic insufficiency (−)	23 %	32.3	21.7
Group 2: shockable rhythm (+), lactate level <9 mmol/L (−), hepatic insufficiency (−)	13 %	22.2	17.4
Group 3: shockable rhythm (−), lactate level <9 mmol/L (+), hepatic insufficiency (−)	10 %	17.9	16.0
Group 4: shockable rhythm (+), lactate level < 9 mmol/L (+), hepatic insufficiency (+)	8 %	14.3	15.1
Group 5: shockable rhythm (−), lactate level < 9 mmol/L (−), hepatic insufficiency (−)	5 %	7.8	13.8
Group 6: shockable rhythm (+), lactate level <9 mmol/L (−), hepatic insufficiency (+)	4 %	4.2	13.2
Group 7: shockable rhythm (−), lactate level <9 mmol/L (+), hepatic insufficiency (+)	3 %	NA	12.7
Group 8: shockable rhythm (−), lactate level <9 mmol/l (−), hepatic insufficiency (+)	2 %	NA	12.0

*CPR* cardiopulmonary resuscitation, *NA* not available (because the probabilities of survival to hospital discharge for group 7 and 8 were below 18 % from the start of CPR)

For secondary outcomes, a lactate level <9 mmol/L was positively associated with a 10-minute ROSC but not significantly associated with neurological outcome (Additional file 5: Table S4).

## Discussion

In this retrospective observational study, results have indicated that the serum lactate level measured during CPR correlated with the outcome of survival to hospital discharge for IHCA. A serum lactate level <9 mmol/L within 10 minutes of CPR initiation was positively associated with survival. Moreover, together with other variables, the serum lactate level could be useful in estimating optimal CPR durations before transition to next strategy for different patient groups, which could help clinicians create further resuscitation strategies in addition to the initial conventional CPR.

Excess production of lactate has been reported to be largely due to tissue hypoxia or hypoperfusion with associated anaerobic metabolism [22]. For the past few decades, it has been noted that the blood lactate level might correlate with the duration of no-flow and low-flow status during CPR [23, 24], both of which are known to impact outcomes after cardiac arrest [25]. This biomarker is therefore a promising candidate for a prognostic predictor of CPR. Weil et al. [11] suggested that the lactate level measured within 10 minutes of CPR could be associated with survival outcomes despite the fact that other important confounders were not considered during their analysis. In subsequent investigations, lower serum lactate measurements or early and effective serum lactate clearance was also shown to correlate with decreased mortality in cardiac arrest patients [12–18]. These studies [12–18] all used post-ROSC lactate level for analysis.

In line with previous studies [11–18], we revealed that the serum lactate level measured during CPR was significantly associated with survival outcomes after adjusting for the confounding effects of multiple factors. There was a near-linear inverse association between the lactate level and the probability of survival. Furthermore, a lactate level <9 mmol/L was significantly associated with higher survival probabilities. This cutoff point was higher than those reported in previous studies [15, 16]. Seeger et al. [15] reported that a lactate level >6.94 mmol/L was associated with poor neurological outcomes; Kaji et al. [16] reported that a lactate level <5 mmol/L correlated with a favourable neurological outcome. The difference in threshold values could be explained by the different time points of lactate measurement and outcome selection. Compared to measurements after ROSC [15, 16], in which blood flow had already recovered to ameliorate systemic hypoxia and hypoperfusion, it seems reasonable that lactate levels would be higher when measured during ongoing CPR.

However, the causes of elevated serum lactate do merit further explanation. Firstly, the serum lactate level could indeed be a surrogate marker for duration of no-flow and low-flow status after cardiac arrest [23, 24]. Nevertheless, Müllner et al. also suggested that the lactate level may be just a weak measure of the duration of cardiac arrest [26]. For IHCA patients, various conditions, such as severe septic shock, could also produce similarly high lactate levels even without cardiac arrest. The use of adrenaline had also been shown to increase the serum lactate level [27]. Secondly, the serum lactate level reflected the difference between lactate production and elimination; thus, an increased lactate level may also indicate mechanisms other than cellular hypoxia or hypoperfusion. For example, impaired liver function could lead to reduced lactate elimination and an elevated lactate level, even when the hemodynamic status was not compromised [28, 29]. Despite these considerations, by adjusting the effects of multiple confounders, including hepatic insufficiency, we believe that the serum lactate level measured during CPR, with its complex physiologic and pathologic implications, could still serve as a useful prognostic factor for outcomes.

There are currently no recommendations for optimal CPR duration before transition to the next strategy [3, 4]. In clinical practice, patients usually received a predetermined duration of CPR, often 30 minutes, even if the patients had not exhibited any response to the repeated resuscitation efforts. However, for adult IHCA patients, Goldberger et al. showed that patients would have a higher probability of survival if they were resuscitated at hospitals that tended to implement longer CPR durations [30]. Furthermore, Matos et al. [10] demonstrated that outcomes after paediatric IHCA could differ significantly between different patient groups, indicating that surgical cardiac patients had the highest probability of a good outcome and trauma patients had the lowest probability, and that CPR >20 minutes may not be futile in certain patient groups. Conversely, Kim et al. [31] recommended that ECPR should be considered for adult patients with out-of-hospital cardiac arrest (OHCA) when CPR was ≥21 minutes. In addition, in an adult OHCA study, Reynolds et al. [32] revealed that the probability of favourable outcomes declined rapidly with each minute of CPR and most surviving patients (89.7 %) with favourable neurological outcomes would achieve ROSC within 16.1 minutes of CPR. Therefore, Reynolds et al. [32] suggested that novel therapeutics, such as ECPR, should be considered early after CPR rather than after the complete failure of resuscitation. This could also hold true for IHCA patients especially for whom applications of ECPR were more available and mature [7].

Using CPR duration alone to define futile resuscitation may risk delaying timely implementation of ECPR and missing opportunities to improve patient outcomes. However, the classification method of patient groups used by Matos et al. [10] was somewhat difficult to be generalized and applied in clinical practice. In our current study, in addition to serum lactate level and CPR duration, we also noted that shockable rhythm and hepatic insufficiency were also significantly associated with survival outcomes, both of which have also been reported in previous studies [33, 34]. Based on these variables, we were able to divide patients into groups with different survival probabilities (Fig. 1). In this way, clinicians may be more prepared for determining the optimal CPR duration for each patient group before transition to the next strategy, either a more advanced strategy, such as ECPR, or termination of CPR.

For example, if we choose the average IHCA survival rate 18 % as a reference [1, 2], we would note that the maximally allowed CPR duration was 32.3 minutes for patient group 1 before the survival probability dropped below 18 %; however, for patient groups 7 and 8, the survival probability would be far below 18 % even from the start of CPR (Table 5). From another perspective, if we choose the commonly used CPR duration of 30 minutes, we could note that even if the CPR intervals were the same, there would be a 10-fold difference in the probability of survival between patient groups 1 (23 %) and 8 (2 %) (Table 5). Therefore, it is clear that if clinicians depended solely on CPR duration to define futile resuscitation, it may be premature to give up for some patients and delayed to activate alternative resuscitation measures for other patients. However, because of the limited number of patients in our analysis, the estimations for CPR duration and survival probability can only serve for the purpose of explanation rather than precise estimations.

A lactate level <9 mmol/L was also significantly associated with a 10-minute ROSC. This CPR interval has been used as one of prerequisites prior to activation of ECPR [7] and termination of IHCA CPR [35, 36]. We suggest that for IHCA patients with a lactate level higher than 9 mmol/L and without exclusion criteria for ECPR, the ECPR team should probably be alerted earlier in order to be able to initiate the ECPR sooner to improve outcomes, as these patients were shown to have less chance of achieving rapid ROSC. Conversely, the clinical decision to terminate IHCA was based solely on witness status, initial arrest rhythm and CPR duration [35, 36]. Using serum lactate level as an additional variable could probably increase the accuracy of this decision and avoid lengthening the dying process of critical patients.

Post-ROSC serum lactate level or the clearance rate of serum lactate has been associated with neurological outcomes [12, 14, 16, 17]. An elevated lactate level may represent a more severe status of tissue hypoperfusion and ischaemia-reperfusion injury, which might aggravate the cerebral dysfunction in post-cardiac arrest syndrome [37]. As there were only 21 patients (6.2 %) in our cohort who regained a favourable neurological outcome, the statistical power may be insufficient to detect the difference between patients with and without a favourable neurological outcome. Nonetheless, the trend of the point estimate of lactate level (odds ratio 1.86) supports our hypothesis. Future studies with more patients would be expected to clarify this issue.

In summary, we found that the serum lactate level measured during CPR was associated with survival outcomes. Along with other variables, the lactate level could help separate patients into several groups with different levels of survival probability. With a predetermined survival probability, clinicians may be able to determine the optimal CPR duration for each group. However, in our current study, we were not aiming to obtain and recommend a fixed CPR duration for each patient group as this was dependent on individual patients. We also did not intend to analyse which strategies should be undertaken following implementation of this optimal CPR duration. What we did emphasize, however, was that the CPR duration alone should not be used for decision-making in CPR. The optimal resuscitation strategy should be personalised and be adjusted to the specific context, taking into account the autonomy of patients, wishes of family members, or the availability of ECPR and other resources of critical care.

### Study limitations

First, this was an observational study, which could only establish an association rather than a causal relationship between independent and dependent variables. Second, the quality of CPR could not be retrospectively determined. Currently, there were no studies indicating that the serum lactate level measured during CPR could reflect the quality of CPR. The association between serum lactate level and quality of CPR should be further examined in a prospective method. Third, we could not be sure whether the serum lactate was from arterial or venous samples. Ralston et al. [23] reported that there was no significant difference in lactate levels between arterial and venous samples during animal CPR. Therefore, this issue may not bias our results to a significant extent. Fourth, the proportion of patients receiving therapeutic hypothermia was low in our cohort. The influence of therapeutic hypothermia on the association between lactate levels and CPR outcomes might be left for future researchers to investigate. Fifth, we used survival to hospital discharge as the primary outcome because the number in the studied cohort was small. Nonetheless, neurologically intact survival might be a more important and meaningful long-term outcome and should be explored as the primary outcome in future research with larger patient populations. Finally, we used IHCA patients for analysis, and so the results might not be applicaple to OHCA patients. In addition, the current analysis was a retrospective study in a single centre with a highly selective cohort, so the probability for selection bias might be high. The lactate levels measured during the initial 10 minutes of CPR were only available in 30 % (340/1123) of the total IHCA patients. Most of the lactate values were reported simultaneously with measurements of blood gases or potassium by point-of-care machines. As resuscitation guidelines [3, 4] do not suggest the measurement of lactate level during CPR, only a prospective study could avoid this kind of selection bias.

## Conclusions

The serum lactate level measured during CPR may correlate with survival outcomes. A 9-mmol/L lactate level could probably be used as a reference value to identify patients with different survival probabilities and to determine the optimal duration of CPR.

## Key messages

- The serum lactate level measured during CPR may correlate with survival outcomes
- A 9-mmol/L lactate level could probably be used as a reference value to identify patients with different survival probabilities and to determine the optimal CPR durations

## Additional files

Additional file 1: Table S1. Definitions of comorbidities used in the multivariable models. (DOCX 21 kb) Additional file 2: Figure S1. Generalized additive model plot for non-parametric modelling of the effect of serum lactate level on the logit of probability for survival to hospital discharge. (TIFF 23 kb) Additional file 3: Table S2. Baseline characteristics of study patients stratified by lactate level. (DOCX 17 kb) Additional file 4: Table S3. Features of cardiac arrest events stratified by lactate level. (DOCX 18 kb) Additional file 5: Table S4. Multiple logistic regression model with secondary outcomes as the dependent variable. (DOCX 17 kb)

## Abbreviations

CPC: cerebral performance category
CPR: cardiopulmonary resuscitation
ECPR: extracorporeal cardiopulmonary resuscitation
GAM: generalized additive model
ICU: intensive care unit
IHCA: in-hospital cardiac arrest
NTUH: National Taiwan University Hospital
OHCA: out-of-hospital cardiac arrest
ROSC: rapid return of spontaneous circulation
