# Supplementary material for: Monitoring of serum lactate level during cardiopulmonary resuscitation in adult in-hospital cardiac arrest
Source: Crit Care. 2015 Sep 21;19(1):344. doi: 10.1186/s13054-015-1058-7 (PMC4576402; doi:10.1186/s13054-015-1058-7)
Supplement: Additional file 1: Table S1. — Definitions of comorbidities used in the multivariable models. (DOCX 21 kb) [file 13054_2015_1058_MOESM1_ESM.docx]

Additional file 1: Table S1. Definitions of Comorbidities Used in the Multivariable Models

| Variables | Definitions |
| --- | --- |
| Heart failure | Documented diagnosis of congestive heart failure during this admission or previous admissions |
| Myocardial infarction | Documented diagnosis of myocardial ischemia (acute coronary syndrome) or myocardial infarction during this admission or previous admissions |
| Arrhythmia | Documented diagnosis of a cardiac arrhythmia |
| Hypotension | Evidence of hypotension within 4 hours up to the time of the event, defined by ANY of the following: 1. SBP < 90 or MAP < 60 mmHg; 2. Vasopressor or inotropic requirement after volume expansion (except for dopamine ≤ 3 mcg/kg/min); 3. Intra-aortic balloon pump |
| Respiratory insufficiency | Evidence of acute or chronic respiratory insufficiency within 4 hours up to the time of the event, defined by ANY of the following  1. PaO2/FiO2 ratio < 300 (in the absence of pre-existing documented cyanotic heart disease)  2. PaO2 < 60 mm Hg (in the absence of pre-existing documented cyanotic heart disease)  3. SaO2 < 90 %, (in the absence of pre-existing documented cyanotic heart disease)  4. PaCO2, EtCO2 or TcCO2 > 50 mm Hg  5. Spontaneous respiratory rate > 40/min or < 5/min  6. Need for non-invasive ventilation (e.g., Bag-Valve-Mask, Mask CPAP or BiPAP, Nasal CPAP or BiPAP, negative pressure ventilation)  7. Need for ventilation via invasive airway (e.g., T-piece, assist control, IMV, pressure support, high frequency) |
| Renal insufficiency | Evidence of renal insufficiency prior to the event, defined by ANY of the following: 1. Requiring ongoing dialysis or extracorporeal filtration therapies; 2. Creatinine > 2 mg/dL within 24 hours up to the time of the event |
| Hepatic insufficiency | Evidence of hepatic insufficiency within 24 hours up to the time of the event, defined by ANY of the following: 1. Total bilirubin > 2 mg/dL and AST > 2x normal; 2. Cirrhosis |
| Metabolic or electrolyte abnormality | Evidence of metabolic/electrolyte abnormality within 4 hours up to the time of the event, defined by ANY of the following: 1. Sodium < 125 or > 150 mEq/L; 2. Potassium < 2.5 or > 6 mEq/L; 3. Arterial pH < 7.3 or > 7.5; 4. Lactate > 2.5 mmol/L; 5. Blood glucose < 60 mg/dL |
| Diabetes mellitus | Documented diagnosis of Type I or Type II diabetes mellitus |
| Baseline evidence of motor, cognitive, or functional deficits  (CNS depression) | Evidence of a motor, cognitive, or functional baseline deficit (at time of system entry) |
| Acute stroke | Documented diagnosis of an intracranial or intraventricular hemorrhage or thrombosis during this admission |
| Favorable neurological status 24 hours before cardiac arrest | Best CPC score within 24 hours before cardiac arrest CPC1: Good cerebral performance: conscious, alert, able to work, might have mild neurologic or psychologic deficit.  CPC2: Moderate cerebral disability: conscious, sufficient cerebral function for independent activities of daily life. Able to work in sheltered environment.  CPC3: Severe cerebral disability: conscious, dependent on others for daily support because of impaired brain function. Ranges from ambulatory state to severe dementia or paralysis.  CPC4: Coma or vegetative state: any degree of coma without the presence of all brain death criteria. Unawareness, even if appears awake (vegetative state) without interaction with environment; may have spontaneous eye opening and sleep/awake cycles.  CPC5: Brain death: apnea, areflexia, EEG silence, etc. |
| Pneumonia | Documented diagnosis of active pneumonia, where antibiotics have not yet been started or the pneumonia is still being treated with antibiotics. |
| Metastatic Cancer or any blood borne malignancy | Any solid tissue malignancy with evidence of metastasis, or any blood borne malignancy |
