# Supplementary figures and images for: Monitoring of serum lactate level during cardiopulmonary resuscitation in adult in-hospital cardiac arrest
Source: Crit Care. 2015 Sep 21;19(1):344. doi: 10.1186/s13054-015-1058-7 (PMC4576402; doi:10.1186/s13054-015-1058-7)

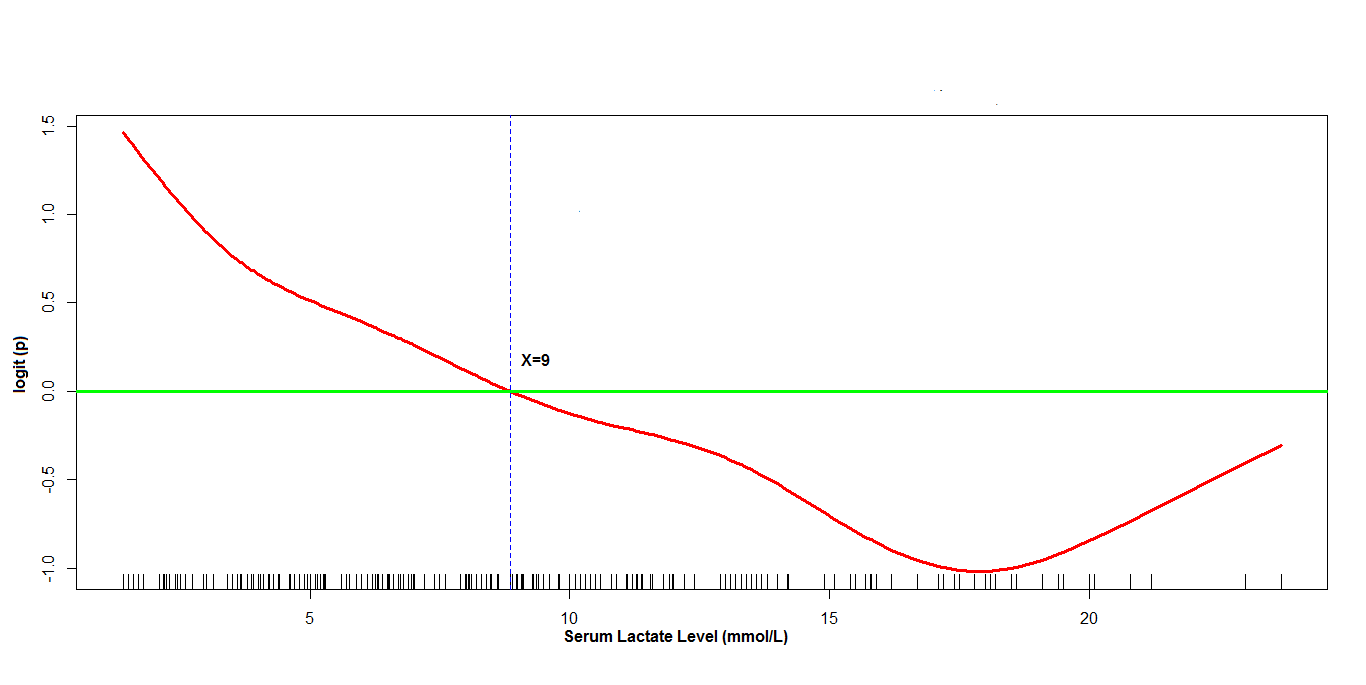

Supplement: Additional file 2: Figure S1. — Generalized additive model plot for non-parametric modelling of the effect of serum lactate level on the logit of probability for survival to hospital discharge. (TIFF 23 kb) [file 13054_2015_1058_MOESM2_ESM.tif]
