# Supplementary material for: Monitoring of serum lactate level during cardiopulmonary resuscitation in adult in-hospital cardiac arrest
Source: Crit Care. 2015 Sep 21;19(1):344. doi: 10.1186/s13054-015-1058-7 (PMC4576402; doi:10.1186/s13054-015-1058-7)
Supplement: Additional file 3: Table S2. — Baseline characteristics of study patients stratified by lactate level. (DOCX 17 kb) [file 13054_2015_1058_MOESM3_ESM.docx]

Additional file 2: Table S2. Baseline characteristics of study patients stratified by lactate level

| Variables | All patients (n = 340) | Lactate level < 9 mmol/L (n = 147) | Lactate level ≧9 mmol/L (n = 193) | *p*-value |
| --- | --- | --- | --- | --- |
| Age, y (SD ^a^) | 65.9 (16.2) | 67.9 (15.2) | 64.3 (16.8) | 0.07 |
| Male, n (%) | 214 (63) | 98 (67) | 116 (60) | 0.26 |
| Comorbidities, n (%) |  |  |  |  |
| Heart failure | 93 (27) | 43 (29) | 50 (26) | 0.54 |
| Myocardial infarction | 45 (13) | 19 (13) | 26 (14) | 1 |
| Arrhythmia | 55 (16) | 30 (20) | 25 (13) | 0.07 |
| Hypotension | 68 (20) | 30 (20) | 38 (20) | 0.89 |
| Respiratory insufficiency | 236 (69) | 103 (70) | 133 (69) | 0.91 |
| Renal insufficiency | 138 (41) | 61 (42) | 77 (40) | 0.82 |
| Hepatic insufficiency | 64 (19) | 21 (14) | 43 (22) | 0.07 |
| Metabolic or electrolyte  abnormality | 66 (19) | 23 (16) | 43 (22) | 0.13 |
| Diabetes mellitus | 112 (33) | 44 (30) | 68 (35) | 0.35 |
| Baseline evidence of motor, cognitive, or functional deficits | 63 (19) | 24 (16) | 39 (20) | 0.40 |
| Acute stroke | 16 (5) | 6 (4) | 10 (5) | 0.80 |
| Favorable neurological status 24 hours before cardiac arrest | 143 (42) | 60 (41) | 83 (43) | 0.74 |
| Pneumonia | 111 (33) | 55 (37) | 56 (29) | 0.10 |
| Metastatic cancer or any blood-borne malignancy | 68 (20) | 22 (15) | 46 (24) | 0.05 |

^a^ SD, standard deviation
