# Supplementary material for: Monitoring of serum lactate level during cardiopulmonary resuscitation in adult in-hospital cardiac arrest
Source: Crit Care. 2015 Sep 21;19(1):344. doi: 10.1186/s13054-015-1058-7 (PMC4576402; doi:10.1186/s13054-015-1058-7)
Supplement: Additional file 4: Table S3. — Features of cardiac arrest events stratified by lactate level. (DOCX 18 kb) [file 13054_2015_1058_MOESM4_ESM.docx]

Additional file 3: Table S3. Features of cardiac arrest events stratified by lactate level

| Variables | All patients (n = 340)(%) | Lactate level < 9 mmol/L  (n = 147) | Lactate level ≧9 mmol/L  (n = 193) | *p*-value |
| --- | --- | --- | --- | --- |
| Arrest at night, n (%) | 218 (64) | 96 (65) | 122 (63) | 0.73 |
| Arrest at the weekend, n (%) | 105 (31) | 46 (31) | 59 (31) | 0.91 |
| Arrest location, n (%) |  |  |  | 0.19 |
| Intensive care unit | 151 (44) | 67 (46) | 84 (44) |  |
| General ward | 171 (50) | 76 (52) | 95 (49) |  |
| Others | 18 (5) | 4 (3) | 14 (7) |  |
| Witnessed arrest, n (%) | 241 (71) | 102 (69) | 139 (72) | 0.63 |
| Monitored status, n (%) | 199 (59) | 87 (59) | 112 (58) | 0.91 |
| Shockable rhythm, n (%) | 49 (14) | 28 (19) | 21 (11) | 0.04 |
| Critical care interventions in place at time of arrest |  |  |  |  |
| Mechanical ventilation | 64 (19) | 25 (17) | 39 (20) | 0.49 |
| Anti-arrhythmics | 30 (9) | 13 (9) | 17 (9) | 1 |
| Vasopressors | 154 (45) | 64 (44) | 90 (47) | 0.58 |
| Dialysis | 23 (7) | 11 (8) | 12 (6) | 0.67 |
| Pulmonary artery  catheter | 2 (0.6) | 2 (1) | 0 (0) | 0.19 |
| Intra-aortic balloon  pumping | 1 (0.3) | 1 (1) | 0 (0) | 0.43 |
| Lactate (mmol/L) (SD^a^) | 9.6 (4.6) | 5.4 (2.0) | 12.8 (3.0) | < 0.001 |
| CPR^b^ duration, min (SD) | 28.8 (26.9) | 23.2 (23.7) | 33.1 (28.5) | < 0.001 |
| Post-ROSC ^c^ interventions, n (%) |  |  |  |  |
| Extracorporeal membrane oxygenation | 20 (6) | 7 (5) | 13 (7) | 0.49 |
| Therapeutic hypothermia | 1 (0.3) | 0 (0) | 1 (0.5) | 1 |
| Percutaneous coronary intervention | 8 (2) | 5 (3) | 3 (2) | 0.30 |

^a^ SD, standard deviation

^b^ CPR, cardiopulmonary resuscitation

c ROSC, return of spontaneous circulation
