# Supplementary material for: Monitoring of serum lactate level during cardiopulmonary resuscitation in adult in-hospital cardiac arrest
Source: Crit Care. 2015 Sep 21;19(1):344. doi: 10.1186/s13054-015-1058-7 (PMC4576402; doi:10.1186/s13054-015-1058-7)
Supplement: Additional file 5: Table S4. — Multiple logistic regression model with secondary outcomes as the dependent variable. (DOCX 17 kb) [file 13054_2015_1058_MOESM5_ESM.docx]

Additional file 4: Table S4. Multiple logistic regression model with secondary outcomes as the dependent variable

| Independent variable | Odds ratio | 95% confidence interval | *p* value^c^ |
| --- | --- | --- | --- |
| *Outcome: 10-min ROSC*^a^ |  |  |  |
| Lactate level < 9 mmol/L | 3.36 | 1.92-6.00 | <0.001 |
| Renal insufficiency | 2.50 | 1.44-4.40 | 0.001 |
| Arrest at intensive care unit | 2.78 | 1.46-5.41 | 0.002 |
| Age | 0.98 | 0.96-0.99 | 0.005 |
| Vasopressors in place at time of arrest | 0.45 | 0.23-0.87 | 0.02 |
| *Outcome: Favorable neurological outcome at hospital discharge* | | | |
| CPR^b^ duration | 0.86 | 0.78-0.93 | <0.001 |
| Shockable rhythm | 5.56 | 1.97-15.75 | 0.001 |
| Lactate level < 9 mmol/L | 1.86 | 0.63-6.25 | 0.28 |

^a^ ROSC: Return of spontaneous circulation

^b^ CPR: Cardiopulmonary resuscitation

^c^ The display of independent variables is arranged in order of *p* value.

^d^ For model of 10-min ROSC: Goodness-of-fit assessment: n = 340, adjusted generalized *R^2^* = 0.19, the estimated area under the Receiver Operating Characteristic (ROC) curve = 0.74, and the modified Hosmer and Lemeshow goodness-of-fit Chi-Squared test *p* = 0.70.

^e^ For model of favorable neurological outcome at hospital discharge: Goodness-of-fit assessment: n = 340, adjusted generalized *R^2^* = 0.359, the estimated area under the Receiver Operating Characteristic (ROC) curve = 0.90, and the modified Hosmer and Lemeshow goodness-of-fit Chi-Squared test *p* = 0.82.
